# Automated Ultra-Fast ^13^C NMR Analysis of Polyolefin Materials

**DOI:** 10.1021/acs.analchem.4c06290

**Published:** 2025-01-21

**Authors:** Fabio Giampaolo, Roberta Cipullo, Salvatore Cuomo, Francesco Piccialli, Vincenzo Busico

**Affiliations:** † Department of Mathematics and Applications “R. Caccioppoli”, University of Naples Federico II, 80126 Naples, Italy; ‡ Department of Chemical Sciences, University of Naples Federico II, 80126 Naples, Italy

## Abstract

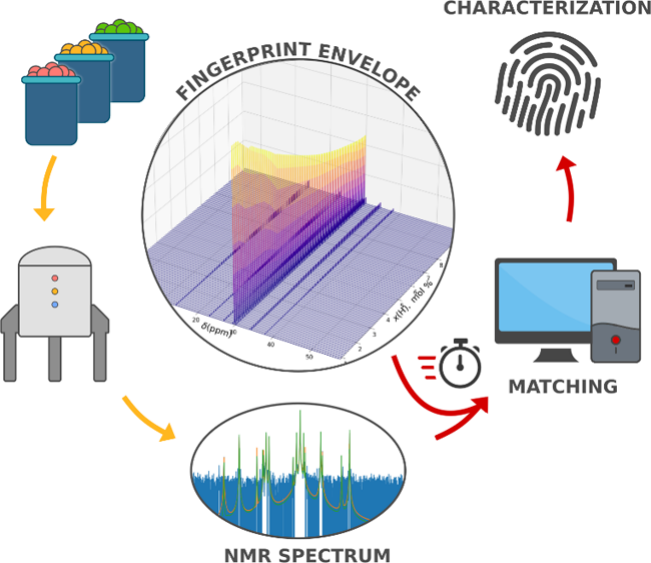

Polyolefins are unique among synthetic polymers because their wide application envelope originates from a finely controlled microstructure of hydrocarbon chains, lacking any distinctive functional groups. This hampers the methods of automated sorting based on vibrational spectroscopies and calls for much more complex ^13^C NMR elucidations. High-temperature cryoprobes have dramatically shortened the acquisition time of ^13^C NMR spectra, and few minutes are now enough for polyolefin classification purposes; however, conventional data analysis remains labor and time-consuming. In this paper, we introduce an instrument for automated fast determinations of the ^13^C NMR microstructure on polyolefin materials, implemented by integrating High-Throughput Experimentation and Data Science tools and methods. From the scientific standpoint, the main interest of the approach is the solution proposed to address the general problem how to rapidly characterize statistically distributed analytes, of which synthetic polymers are a most important case. In practical terms, the instrument represents the first automated tool for microstructural polyolefin analysis: it is readily applicable to monomaterials, whereas extension to multimaterials, including postconsumer streams, is feasible but still requires some work.

## Introduction

A sustainable society needs plastics as much as practical ways to recycle plastic wastes.^1−4^ Redesign the market of virgin products privileging monomaterials, enforce a separate collection of postconsumer products, sort any residual multimaterial wastes and implement economically viable methods for mechanical or thermal recycling are complementary and equally important actions of a comprehensive strategy that must be given utmost priority.

In recent years major progress has been achieved, and for certain plastics (like e.g. polyesters and polyamides) the fraction of recycled wastes is approaching that of paper and some common metals.^1,2,4^ However, in a generally positive scenario polyolefins lag behind,^4^ which is truly unfortunate because altogether polyethylene (PE) and polypropylene (PP) materials represent roughly 50% by weight of all produced plastics (about 200 million metric tons in 2023).^2,5,6^ What makes polyolefins unique is that their wide properties envelope stems from precisely controlled distributions of monomeric units lacking any functional groups. The aliphatic hydrocarbon nature determines a high chemical and environmental inertness, which is a formidable asset for most applications but also a severe drawback for postconsumer sorting and reutilization purposes; in particular, the comparatively featureless vibrational spectra of polyolefins limit the scope of automated sorting of waste streams based on Near-IR (NIR) spectroscopy,^7,8^ that can be used at most to discriminate PE from PP. Ironically, the chemical similarity of PE and PP, while complicating analytical tasks, does not result into thermodynamic compatibility of mixtures,^9^ and with the only exception of some finely dispersed reactor blends (like e. g. “High-Impact PP”, see below) phase-separated polyolefin blends have limited, low value-added applications. The addition of compatibilizers,^10^ now commercially available at affordable prices (e. g., block copolymers produced by tandem catalysis under “chain shuttling” conditions^11^), can mitigate the problem and facilitate mechanical recycling, but the wealth of information at molecular level that is needed to design high performance polyolefin multimaterials is beyond the reach of simple (and inexpensive) analytical tools.

Thorough determinations of polyolefin microstructure are only feasible by means of ^13^C Nuclear Magnetic Resonance (NMR) spectroscopy.^12,13^ Compared with NIR and even ^1^H NMR, ^13^C NMR data acquisition is technically more complex and time demanding; however, with modern high-temperature cryoprobes the process can be accomplished in few minutes.^14−16^ Quantitative ^13^C NMR spectra of polyolefin monomaterials readily provide access to the relative amounts and sequence distributions of constitutional and configurational units.^12,13,17^ For multimaterials, on the other hand, the inherently limited resolution of the spectra results into extensive resonance overlaps denying access to important parts of the information. In all cases, data analysis with conventional methods is time and labor intensive, and as such unsuited for high throughput screenings. In this paper we introduce an original Data Science (DS) aided approach laying the foundation for automated ultrafast ^13^C NMR analyses of polyolefins. The method is immediately applicable to monomaterials; extension to virgin and postconsumer mixtures is in progress, as explained in the final section.

## Experimental Section

### Synthesis and ^13^C NMR Characterization of the Polyolefin Materials

All olefin polymerization experiments were performed in a Freeslate Parallel Pressure Reactor (PPR) setup with 48 reaction cells, fully contained in a triple MBraun glovebox operating under nitrogen. Full details on the setup and operating protocols were reported before.^18−21^ The polyolefin samples were characterized by means of quantitative ^13^C NMR spectroscopy using a Bruker DRX 400 setup equipped with a high-temperature cryoprobe for 5 mm OD tubes and a preheated robotic sample changer. The spectra were taken sequentially with automated tuning, matching, and shimming. Acquisition conditions were: 45° pulse; acquisition time, 2.7 s; relaxation delay, 5.0 s; 2 K transients. Broad-band proton decoupling was achieved with a modified WALTZ16 sequence (BI_WALTZ16 32 by Bruker). Conventional determinations of sample composition were carried out according to known literature methods.^22^

### Fingerprint Extraction

The quantitative ^13^C NMR spectrum of any polyolefin material, be it known or unknown, undergoes a meticulous processing procedure to extract a signal referred to as “Fingerprint” (FP). Initial preprocessing steps of denoising, baseline correction and smoothing are carried out to minimize artifacts arising from the data collection process, thereby ensuring the reliability and accuracy of subsequent analyses. In the denoising step, a discrete wavelet transform is applied to the spectrum (DWT)^23^ with a Haar wavelet function. The noise level is estimated from the wavelet coefficients, and a threshold is applied to remove noise through soft thresholding. The denoised signal is then reconstructed by applying the inverse wavelet transform. Following the denoising procedure, a baseline correction is performed: this involves identifying noise regions at both ends of the spectrum and fitting a polynomial on them.^24^ This polynomial baseline is then subtracted from the original spectrum to correct for any drift. To further enhance the signal, a Savitzky-Golay filter^25^ is applied, which smooths the spectrum while preserving its features. The mean and standard deviation of the smoothed spectrum are calculated to set a noise threshold, above which the signal is retained. The preprocessing procedure just described ensures the extraction of a meaningful FP containing all and only the relevant information necessary for analytical purposes.

To obtain a FP that is truly representative of the spectrum, the signal is processed approximating each peak in the spectrum using Voigt profiles,^26^ which combine the characteristics of Gaussian and Lorentzian functions to accurately represent the shape of each spectral peak. To this aim the reconstructed spectrum *S̃* (λ) can be intended as composed by a sum of these functions:
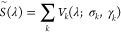
1where *V*_*k*_(λ; σ_*k*_, γ_*k*_) represents the Voigt profile for the *k*-th peak. The parameters σ_*k*_ and γ_*k*_ are optimized with respect to the difference between the reconstructed and preprocessed spectra through the minimization of a designed loss function which combines two key components: the area difference and the shape difference between the original and reconstructed spectra.

The first component, *L*_a_, minimizes the area difference:
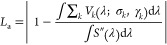
2while the second component, *L*_s_, focuses on the shape difference, using a logarithmic scale to emphasize discrepancies in peak shapes:
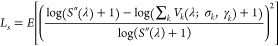
3

The overall objective function, *L*, can be expressed as a weighted sum of these two components:
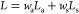
4where *w*_a_ and *w*_s_ are weights that balance the importance of the area and shape differences. The result of this process is an analytical replica of the original spectrum that best fits the data while preserving the essential features of the peaks. This reconstructed spectrum serves as the basis for the subsequent analyses, including the construction of “Fingerprint Envelopes” (see below).

### Fingerprint Envelope (FPE) Construction for Copolymer Monomaterials

Libraries of discrete FP’s for monomaterial copolymer samples at variable composition belonging in each (sub)class of Table 1 were utilized to create a continuous 2D function, that we denominated “Fingerprint Envelope” (FPE), modeling the evolution of the FP with composition. This function has several key utilizations:

1. It can be used to extract the FP of any individual sample in a given (sub)class within the set composition limits.
2. In the opposite direction, it allows to determine the composition of any individual sample in the (sub)class by matching the experimental FP with synthetic replicas within the FPE, according to a procedure described in detail below.
3. In case of monomaterials (demonstrated to be so by an independent method such as e.g. GPC) whose nature is unknown, the matching procedure can be executed scanning the entire FPE archive and to deliver both chemical identity and composition.
4. Last but not least, the FPE representation is amenable to data augmentation techniques.

**Table 1:** Proposed Microstructural Categorization of Commercial Polyolefin Materials

class	sub-class	nature	composition^a^	notes
high density polyethylene (HDPE)		homopolymer		pure high-molar-mass HDPE samples ideally feature one single ^13^C NMR resonance
linear-low-density polyethylene (LLDPE)	E/B-LLDPE	copolymer	*x*(B) < 10%	random copolymers of ethene (E) and 1-butene (B)
	E/H-LLDPE	copolymer	*x*(H) < 10%	random copolymers of ethene (E) and 1-hexene (H)
	E/O-LLDPE	copolymer	*x*(O) < 10%	random copolymers of ethene (E) and 1-octene (O)
low-density polyethylene (LDPE)		homopolymer		the type and distribution of short and long side-chain branches is variable, mainly depending on the process
isotactic polypropylene (iPP)		homopolymer		if desired, the degree of stereoregularity can be added as a microstructural feature
				PP samples made with heterogeneous Ziegler–Natta catalysts are monomaterials only in a first approximation (see text)
raco-PP		copolymer	*x*(E) <10%	random isotactic copolymers of propene (P) with ethene (E)
				the degree of stereoregularity of the PP homosequences can be added as a microstructural feature
ethylene/propylene rubber (EPR)		copolymer	40% < *x*(E) < 60%	random copolymers of ethene (E) and propene (P)
				reactor blends of iPP and EPR are commercially known as “high-impact PP” (HIPP)

a *x*(Y) = mole fraction of (co)monomeric units Y. N/A = not applicable.

To construct the FPE from a proper library of FP’s we employ a robust interpolation technique ensuring that the FPE accurately represents the continuous variation of the spectrum–and correspondingly of the FP–with varying composition, and addressing at the same time the issue of potential artifacts from both the analytical reconstruction and the interpolation itself. Taken the fingerprints related to a specific PO (sub)class, a random subset  from the data set  of analytic reconstructions is selected. This subset is chosen to be half the size of the complete data set:
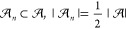


Next, we fit an interpolation model *f*_*n*_(λ, *m*) to  for each iteration. This two-step process is repeated multiple times to account for the variability and ensure robustness. The final interpolation model *f*(λ, *m*) is obtained by fitting a Bivariate Spline on the data set obtained over *N* iterations:
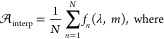

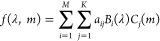
where *B*_*i*_(λ) and *C*_*j*_(*m*) are spline basis functions, and *a*_*ij*_ are the coefficients optimized to fit .

### Matching Procedure

The principal task of this phase involves developing a robust methodology to match an experimental FP with a database of known ones for accurate identification and quantitation purposes. Recall that from the “Fingerprint Envelopes”, it is possible to extract the fingerprint of any member of a PO (sub-) class with a chosen microstructure, provided that the microstructure falls within the interval defined by the minimal and maximal compositions of spectra used to build the Envelope. Given an experimental spectrum, the key steps in this procedure involve first extracting its related experimental fingerprint. This object is represented by a preprocessed spectrum where only the significant parts are preserved, corresponding to the stage before the analytical reconstruction in the preprocessing phase. The next step is to compare this experimental fingerprint with the fingerprints available in our database using a reliable and efficient matching function capable of handling the variability inherent in experimental data.

In particular, let *S̃* be the generic fingerprint extracted from the Envelope and *S*^′′^ be the fingerprint of the experimental spectrum. The distance between the two signals is evaluated in terms of the area under the curve within defined domains. This is achieved with a two-components distance function that measures the differences in area distribution under the significant peaks of the spectra. For corresponding peaks (peaks with the same positions), the area difference should be minimal, and all peaks should overlap for perfect matching. The first term of this distance function, the Peak Domain Difference, is calculated by evaluating the Frobenius norm of the differences across all peak domains:
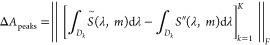
5where *K* is the total number of peak domains, and *D*_peaks_ = ∑_*K*_*D*_*k*_ represents the regions where *S*′′ (λ, *m*) > 0. The second measure, the Residual Domain Difference, is similarly calculated:
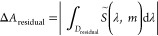
6where the residual domain *D*_residual_ encompasses areas without significant peaks. The combined metric Δ*A*_peaks_ + Δ*A*_residual_ quantifies the discrepancy between the “synthetic” and the experimental signal.

## Results and Discussion

### General Considerations and Brief Illustration of the Analytical Workflow

Machine Learning and Deep Learning applications in NMR are numerous, and a number of well-working protocols have been reported for the identification and quantitation of small molecules (either neat or in mixtures), as well as for structural elucidations of biomacromolecules.^27−30^ The case of synthetic macromolecules, on the other hand, is conceptually and practically different because molecular structure is statistically distributed, and chain properties like molar mass or microstructure can only be quantified as averages that can take any values within more or less wide ranges. In practice, for polyolefin resins this means that no two samples have identical microstructures.^13^

**Figure 1:**
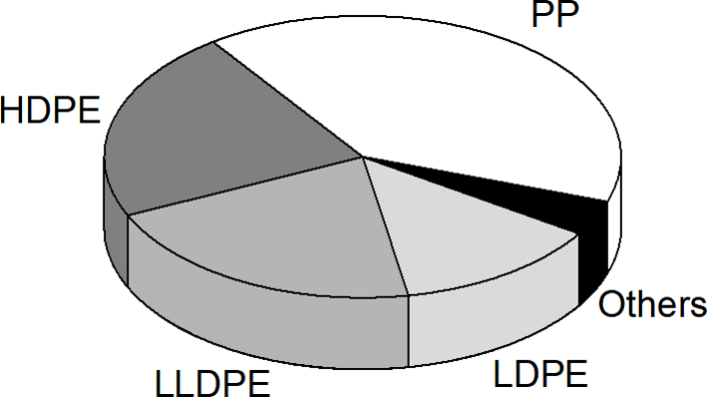
Polyolefin market shares (by weight)^1^: High-density PE (HDPE), 22%; linear-low-density PE (LLDPE), 21%; low-density PE (LDPE), 13%; PP, 40%; others, 5%. The share of PP includes the isotactic homopolymer, random copolymers, and “high-impact” PP (HIPP). See text and Table 1.

On the other hand, polyolefin monomaterials are amenable to a rather simple chemical and microstructural categorization (Table 1). Although the table is not exhaustive, it can be safely stated that it covers >90% by weight of all virgin and postconsumer polyolefins on the market (Figure 1).

For each (sub)class in Table 1, the ^13^C NMR spectrum represents a diagnostic “fingerprint” (short notation “FP”) consisting of a unique set of resonances. In the case of homopolymers, in a first approximation, the fingerprint is univocal. In that of copolymers, instead, relative resonance integrals in the set are a nonlinear function of chemical composition, which is a continuous variable. The literature teaches how to assign the resonances and determine sample composition from their integrals.^22^ In principle, the inverse procedure can be utilized to calculate the spectra of copolymer samples at any composition; in practice, however, the task is complex and impractical because the various resonances feature different resolutions (from the minimum level of constitutional diads up to hexads or even higher).

Aiming to implement an automated DS-aided analytical tool we opted for an empirical approach leveraging the High Throughput Experimentation (HTE) infrastructure of our laboratory.^18,31^ In brief, our protocol was as follows (for full details see Experimental Section):

a) Adequately large libraries of copolymer samples at variable composition in the commercially relevant range of all copolymer (sub)classes were prepared with competent catalysts;
b) Quantitative ^13^C NMR spectra of all samples in each library were recorded under identical conditions and converted into a discrete collection of digital FP’s
c) A continuous function, that we denominated “Fingerprint Envelope” (short notation “FPE”), was constructed by interpolation of the discrete FP’s in each library;
d) A mathematical procedure was implemented for matching the experimental FP of *any* sample in a given (sub)class with its synthetic replica in the FPE.

Automatically executing step (d) over the entire FPE portfolio, added with the univocal FP’s of the homopolymers, returns the microstructural ^13^C NMR analysis of any *unknown* polyolefin monomaterial belonging in the (sub)classes of Table 1; the process only takes few seconds.

In view of the additivity of ^13^C NMR spectra, the approach can be extended to multimaterials, which are a significant fraction of the virgin polyolefin market and practically the entirety of postconsumer streams. However, long computational times and large covariance-related errors in quantitative applications due to the aforementioned limitations in spectral resolution represent major drawbacks. A Deep Learning (DL) tool making use of Neural Network (NN) architectures is a more convenient option; a first perspective account of this part, which is still work in progress, is provided in the last part of this section.

### Automated Analysis of Polyolefin Monomaterials

Qualitatively, the concept of ^13^C NMR FP is general and holds for any polyolefin monomaterial in Table 1, irrespective of whether it is a homopolymer or a copolymer; quantitatively, however, the difference between the two cases is profound. As already noted above, the FP of a homopolymers is univocal at a level of description that disregards the inevitable presence of defects^13^; whereas such defects do have important effects on material properties, they can be ignored for the purpose of this study, at least in a first approximation. The case of LDPE, with its complex branch-on-branch architecture resulting from radical polymerization, is peculiar because relative resonance integrals depend on the process, but their chemical shifts are idiosyncratic and as such adequate for identification purposes.^22^ Copolymer chains, instead, are made of two or more comonomeric units, and FPE’s are mandatory to account for the continuously distributed value(s) of average composition.^22^ Importantly, we found out that catalyst-related differences in comonomer sequence distributions at a given composition are inconsequential in the execution of the FP matching process.

FPE’s for the five (sub)classes of copolymers in Table 1 (namely: E/B-, E/H- and E/O-LLDPE; EPR; raco-PP) were built from libraries of digital FP’s extracted from the ^13^C NMR spectra of copolymers at variable composition by means of a robust interpolation procedure (see Experimental Section). The complete workflow is highlighted in the main text for the subclass of E/H-LLDPE materials, chosen as a representative example. For E/B-LLDPE, E/O-LLDPE, EPR and raco-PP materials we refer to the Supporting Information (SI) file (Tables S3–S7 and Figure S1).

**Figure 2:**
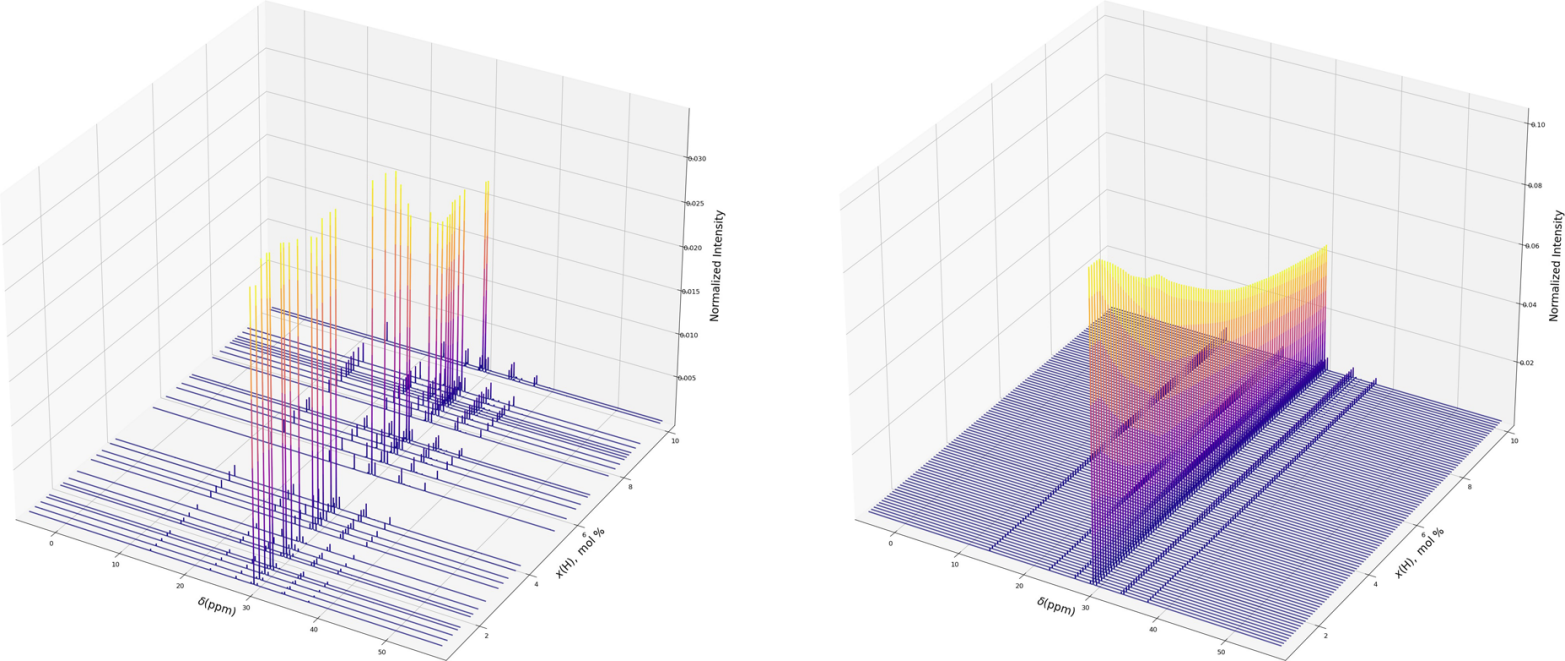
Experimental FP’s of the E/H-LLDPE samples in Table S1 (left) and interpolating E/H-LLDPE FPE (right).

Thirty-two E/H-LLDPE samples with compositions in the commercially relevant range (Table S1) were prepared in a HTE polymerization platform using zirconocene catalysts and analyzed conventionally by quantitative ^13^C NMR spectroscopy in solution (see Experimental Section). The discrete FP’s extracted from the spectra and the continuous interpolating FPE function are shown in Figure 2.

**Figure 3:**
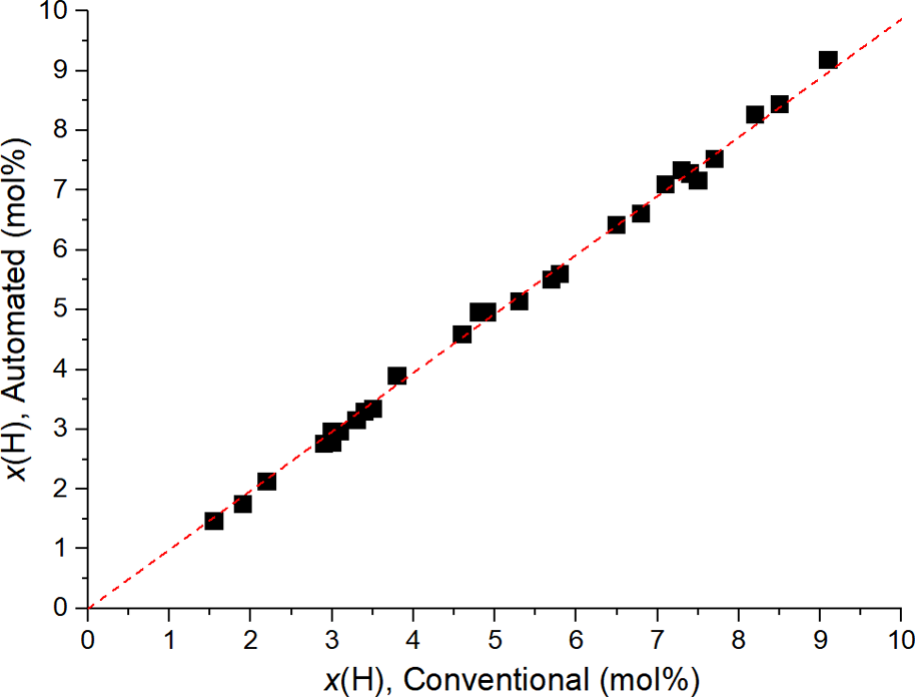
Correlation plot between conventional and automated measurements of composition for the E/H-LLDPE samples of Table S2.

Automated determinations of composition for a validation library of 30 more E/H-LLDPE samples (Table S2) gave very good results, as the correlation plot in Figure 3 demonstrates.

### Polyolefin Multimaterials

Rigorously speaking, all virgin polyolefin grades on the market produced with heterogeneous catalyst systems and/or in reactor cascades are mixtures. In several cases, though, their complex nature cannot be appreciated from ^13^C NMR microstructure; in particular, homopolymers with unimodal or multimodal molar mass distributions are microstructurally indistinguishable. Propene homopolymers made with heterogeneous Ziegler–Natta catalysts are also a peculiar case: whereas they always contain a minor amount of poorly stereoregular (“atactic”) PP chains along with the largely predominant “highly isotactic” PP ones,^13,32,33^ the ^13^C NMR spectra are deceptively similar to those of true iPP monomaterials made with molecular catalysts, to which for the scope of the present study the extracted FP’s can be approximated. In a general case, though, the ^13^C NMR spectrum of a polyolefin mixture (multimaterial) can be described as the weighted sum of the spectra of individual components. The overall FP_mix_ can be extracted like for a monomaterial, and reproduced synthetically according to the following expression:
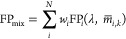
7where *N* is the total number of components, λ is the frequency domain of the spectrum, and FP_*i*_(λ, *m̅*_*i*, *k*_) represents the FP of the *i*^th^-component.

**Figure 4:**
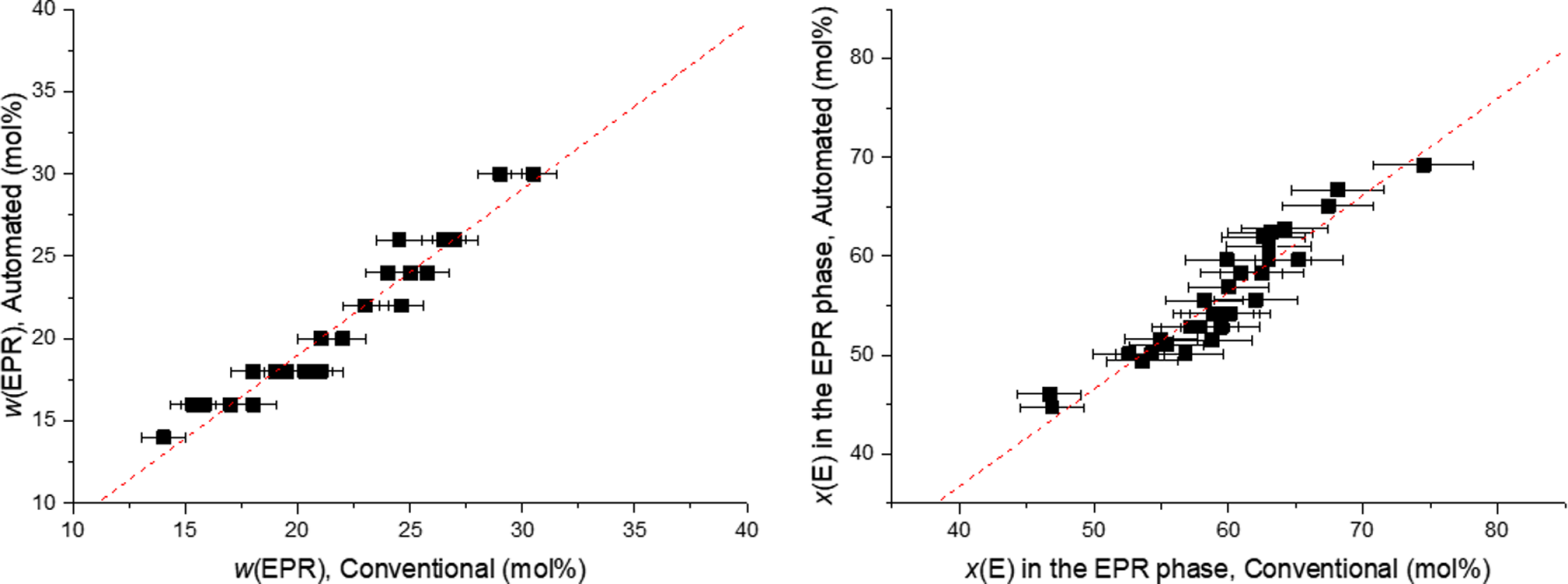
Correlation plots between conventional and automated measurements of composition for the HIPP samples of Table 2 (see text).

Virgin multimaterials usually consist of only few components of known nature, and applying eq 1 for analytical purposes is relatively straightforward. Heterophasic reactor blends of iPP and EPR, commercially known as “High-Impact PP” (HIPP)^34^ (Table 1, see note in the last row), are a convenient example combining high commercial relevance with ease of approach: indeed, as noted before, in a first approximation the univocal FP of iPP can be used for Ziegler–Natta PP too, and the only unknowns in eq 1 are the weight *w*(EPR) and the composition *x*(E) of the EPR component. Application to a test set of 30 HIPP samples (Table 2) gave very nice results, as illustrated by the correlation plots in Figure 4.

**Table 2:** Library of HIPP Samples Utilized to Test the FP Matching Procedure Based on Eq 1 (See Text)^a^

sample #	*w*(EPR), %	*x*(E), mol %	sample #	*w*(EPR), %	*x*(E), mol %	sample #	*w*(EPR), %	*x*(E), mol %
HIPP-T1	23	60	HIPP-T11	19	57	HIPP-T21	17	65
HIPP-T2	27	62	HIPP-T12	24	60	HIPP-T22	20	62
HIPP-T3	22	63	HIPP-T13	21	59	HIPP-T23	18	67
HIPP-T4	23	63	HIPP-T14	17	59	HIPP-T24	19	47
HIPP-T5	25	60	HIPP-T15	19	55	HIPP-T25	25	47
HIPP-T6	25	61	HIPP-T16	27	55	HIPP-T26	17	53
HIPP-T7	22	63	HIPP-T17	21	63	HIPP-T27	15	54
HIPP-T8	27	58	HIPP-T18	14	74	HIPP-T28	16	54
HIPP-T9	30	57	HIPP-T19	17	68	HIPP-T29	25	59
HIPP-T10	18	58	HIPP-T20	21	64	HIPP-T30	29	59

a The values of *w*(EPR) and *x*(E) were determined by conventional ^13^C NMR methods.^35^

Postconsumer mixtures, on the other hand, represent a much more complex analytical challenge because both the number and the identity of the components are typically unknown. eq 1 may still be used to unravel the composition of a mixture as that corresponding to the best match between experimental and calculated FP_mix_, e. g. based on the cost function described in the Experimental Section (eq 5): calculations for all possible mixtures with a defined number of components *N* should be carried out so as to find out the set of FP_*i*_(λ, *m̅*_*i*, *k*_) and *w*_*i*_ corresponding to the minimum distance. A grid search is probably more advisible than a minimization procedure, due to the nonconvexity of the cost function and because, in case of very unbalanced mixtures with components present in very high and very low relative amounts, strong covariance effects may generate many local minima in which minimization algorithms easily happen to get trapped.

On the other hand, a high-resolution grid (required for accurately scanning the variables space) boosts processing time to several hours for comparatively simple mixtures already. Whatever the choice, we conclude that the approach is impractical.

NN models can offer a convenient, albeit nontrivial alternative. Developing a NN capable of identifying the monomaterial components of a polyolefin multimaterial from the respective FP’s is the necessary first step; successfully exploiting this task would restrict the matching procedure to the relevant FPE’s only, thus strongly reducing the computational demand. On the other hand, moving such a step is a real challenge: in fact, the neural structures must be able to focus on the informative parts of the spectrum (i.e., peak integrals and positions) and distinguish relevant features from background noise. This includes extracting general patterns from the peculiarities of random instrumental variations (such as e.g. white noise and chemical shift drifts), notwithstanding extensive peak superpositions. Attention layers, particularly those developed within the broader framework of transformer architectures, appear to be well-suited to the task; these structures offer the potential to accurately capture the necessary spectral features, provided that they are trained on adequately large amounts of data. The latter condition can be fulfilled by augmenting the experimental FPE’s in the monomaterials portfolio with virtually any numbers of synthetic FP’s; then, framing the problem as a classification task, the NN can be trained to recognize the presence or absence of a certain monomaterial FP in the FP_mix_ of a multimaterial. Preliminary tests carried out on all five (sub-) classes of copolymer monomaterials in our archive ended up with excellent results, thus demonstrating that the approach is feasible.

## Conclusions and Outlook

^13^C NMR spectroscopy is the most powerful technique for *quantitative absolute* elucidations of polyolefin microstructure; however, technical complexity, lengthy operation and high costs limit routine applications with virgin materials in industrial practice, where quality controls can be operated with faster and simpler *relative* characterization methods (albeit often downstream of ^13^C NMR calibrations). Ironically, it is for mixed polyolefin wastes, with their extreme variability of composition, that the analytical power of ^13^C NMR is unrivaled, and automated ultrafast determinations of ^13^C NMR microstructure would enable superior mechanical recycling solutions. Modern R&D approaches to polyolefin catalysis and materials science, where the quest for more sustainable products and processes calls for rapid innovation, can also greatly benefit from such methods.

In the previous sections we noted how DS applications to polyolefin ^13^C NMR analysis must preliminarily address the formidable complication represented by the statistical nature of synthetic polymers (of which polyolefins are a most important class), and introduced a novel integrated HTE/DS approach to the problem. Moving from a basic categorization of polyolefin monomaterials according to chemical structure (Table 1), we defined two key concepts and the corresponding mathematical expressions, namely the ^13^C NMR “Fingerprint” (FP) and “Fingerprint Envelope” (FPE). The ^13^C NMR spectrum of a polyolefin monomaterial is unique, and its digital representation is an idiosyncratic FP that univocally defines chemical identity and composition (in case of copolymers). The latter, though, is a continuous, statistically distributed variable whose description requires a bidimensional mathematical function, that we denominated FPE with the word “Envelope” meant to indicate that it contains all possible FP’s of a given polyolefin (sub)class. Access to state-of-the-art HTE tools and methods enabled us to generate libraries of polyolefin monomaterial samples at variable composition for all copolymer (sub)classes in Table 1, and from the corresponding *discrete* FP’s to construct the *continuous* FPE’s by means of an interpolative method. We then implemented a matching algorithm to scan the FPE portfolio, including homopolymers and copolymers, and locate the synthetic replica of any experimental FP, ending up with the identification and (for copolymers) the exact composition of the corresponding sample.

To the best of our knowledge, this workflow represents the first automated tool reported in the literature for the fast ^13^C NMR analysis of polyolefin monomaterials. Application only takes few seconds, to be compared with several minutes (or more) for conventional elaborations of ^13^C NMR data. Moreover, hyphenation with the operating software of the spectrometer can lead to very substantial reductions of acquisition time, because acquisition can be automatically discontinued as soon as the DS tool is able to finalize the analysis within a desired accuracy.

Extension to multimaterials, on the other hand, is more complex. As repeatedly noted, the ^13^C NMR spectra of polyolefin mixtures suffer from extensive resonance overlaps, and unraveling FP_mix_ functions in terms of summations of monomaterial FP’s leads to solutions with large covariance-related errors and can take an impractically long computational time. We have achieved preliminary indications that an approach based on a NN model bears much more promise, as will be reported in due course.

## Data Availability

The data sets generated and/or analyzed during the current study and the source code developed are available from the corresponding authors on reasonable request.

## Supporting Information Available

^13^C NMR characterizations of monomaterial copolymer samples utilized for FP extraction and FPE construction (PDF)
