# Supplementary material for: Automated Ultra-Fast 13C NMR Analysis of Polyolefin Materials
Source: Anal Chem. 2025 Jan 21;97(4):2503–10. doi: 10.1021/acs.analchem.4c06290 (PMC11801656; doi:10.1021/acs.analchem.4c06290)
Supplement: Supplementary file 1 — ac4c06290_si_001.pdf [file ac4c06290_si_001.pdf]

Supporting Information

Automated Ultra-Fast ^{13}C NMR Analysis of Polyolefin Materials

Fabio Giampaolo,† Roberta Cipullo,*,‡ Salvatore Cuomo,† Francesco Piccialli,*,† and Vincenzo Busico‡

†University of Naples Federico II, Department of Mathematics and Applications "R. Caccioppoli", Italy

‡University of Naples Federico II, Department of Chemical Sciences, Italy

Corresponding authors: rcipullo@unina.it , francesco.piccialli@unina.it

Table of Content

^{13}C NMR characterizations of mono-material copolymer samples utilized for FP extraction and FPE construction:

Table S1. Library of E/H-LLDPE samples. **S3**

Table S2. Library of E/H-LLDPE samples prepared and utilized to test the FP matching procedure inside the E/H-LLDPE FPE. **S3**

Table S3. Library of E/B-LLDPE samples **S4**

Table S4. Library of E/O-LLDPE samples **S4**

Table S5. Library of EPR samples **S5**

Table S6. Library of raco-PP samples **S5**

Table S7. ^{13}C NMR compositions of E/B-LLDPE, E/O-LLDPE, EPR and raco-PP samples utilized to validate the FP matching procedure with the proper FPE's. **S6**

Figure S1. Correlation plots between conventional and automated ^{13}C NMR measurements of composition for the E/B-LLDPE (top left), E/O-LLDPE (top right), EPR (bottom left) and raco-PP (bottom right) samples of Table S7. **S7**

^{13}C NMR characterizations of mono-material copolymer samples utilized for FP extraction and FPE construction.

Table S1. Library of E/H-LLDPE samples.

Sample #	*x*(H), mol %	Sample #	*x*(H), mol %	Sample #	*x*(H), mol %	Sample #	*x*(H), mol %
E/H-1	1.1	E/H-9	3.1	E/H-17	6.3	E/H-25	8.5
E/H-2	1.3	E/H-10	3.3	E/H-18	6.5	E/H-26	8.5
E/H-3	1.5	E/H-11	3.5	E/H-19	6.8	E/H-27	8.6
E/H-4	1.6	E/H-12	3.8	E/H-20	6.9	E/H-28	8.6
E/H-5	2.0	E/H-13	4.0	E/H-21	7.7	E/H-29	8.7
E/H-6	2.1	E/H-14	4.4	E/H-22	8.0	E/H-30	8.9
E/H-7	2.3	E/H-15	5.4	E/H-23	8.2	E/H-31	9.1
E/H-8	2.6	E/H-16	5.9	E/H-24	8.4	E/H-32	10.0

Table S2. Library of E/H-LLDPE samples prepared and utilized to test the FP matching procedure inside the E/H-LLDPE FPE.

Sample #	*x*(H), mol%	Sample #	*x*(H), mol%	Sample #	*x*(H), mol%
E/H-T1	1.55	E/H-T11	3.4	E/H-T21	6.8
E/H-T2	1.55	E/H-T12	3.5	E/H-T22	7.1
E/H-T3	1.9	E/H-T13	3.9	E/H-T23	7.3
E/H-T4	2.2	E/H-T14	4.1	E/H-T24	7.4
E/H-T5	2.9	E/H-T15	4.8	E/H-T25	7.5
E/H-T6	3.0	E/H-T16	5.1	E/H-T26	7.7
E/H-T7	3.0	E/H-T17	5.3	E/H-T27	8.1
E/H-T8	3.1	E/H-T18	5.5	E/H-T28	8.4
E/H-T9	3.1	E/H-T19	5.6	E/H-T29	8.5
E/H-T10	3.3	E/H-T20	6.4	E/H-T30	9.1

Table S3. Library of E/B-LLDPE samples
.

Sample #	x(B), mol%	Sample #	x(B), mol%	Sample #	x(B), mol%
EB-1	0.6	EB-12	1.9	EB-23	6.6
EB-2	0.8	EB-13	2.4	EB-24	6.7
EB-3	0.9	EB-14	2.4	EB-25	7.5
EB-4	1.0	EB-15	2.5	EB-26	8.2
EB-5	1.1	EB-16	3.0	EB-27	8.5
EB-6	1.2	EB-17	3.1	EB-28	8.8
EB-7	1.2	EB-18	3.2	EB-29	9.0
EB-8	1.2	EB-19	3.4	EB-30	9.0
EB-9	1.4	EB-20	3.8	EB-31	9.1
EB-10	1.4	EB-21	5.1	EB-32	9.2
EB-11	1.5	EB-22	6.5	EB-33	10.2

Table S4. Library of E/O-LLDPE samples

Sample #	x(O), mol%	Sample #	x(O), mol%	Sample #	x(O), mol%
EO-1	0.56	EO-11	3.3	EO-21	5.9
EO-2	0.8	EO-12	3.6	EO-22	6.6
EO-3	1.2	EO-13	3.9	EO-23	6.6
EO-4	1.4	EO-14	4.0	EO-24	6.6
EO-5	2.2	EO-15	4.3	EO-25	6.8
EO-6	2.2	EO-16	4.7	EO-26	7.1
EO-7	2.5	EO-17	4.8	EO-27	7.3
EO-8	2.8	EO-18	5.1	EO-28	7.7
EO-9	3.0	EO-19	5.6	EO-29	8.8
EO-10	3.1	EO-20	5.8	EO-30	9.2
		EO-31	9.2		

Table S5. Library of EPR samples

Sample #	x(E), mol%	Sample #	x(E), mol%	Sample #	x(E), mol%
EPR-1	29.7	EPR-11	50.5	EPR-21	62.9
EPR-2	32.3	EPR-12	51.6	EPR-22	63.7
EPR-3	32.5	EPR-13	53.3	EPR-23	64.6
EPR-4	34.3	EPR-14	57.2	EPR-24	64.8
EPR-5	36.9	EPR-15	57.9	EPR-25	65.0
EPR-6	37.1	EPR-16	58.4	EPR-26	65.8
EPR-7	38.0	EPR-17	60.2	EPR-27	66.3
EPR-8	46.0	EPR-18	60.7	EPR-28	68.9
EPR-9	47.3	EPR-19	60.9	EPR-29	69.2
EPR-10	48.3	EPR-20	62.1		

Table S6. Library of raco-PP samples

Sample #	x(E), mol%	Sample #	x(E), mol%	Sample #	x(E), mol%
raco-PP-1	1.8	raco-PP-PP-9	3.0	raco-PP-18	5.0
raco-PP-2	2.2	raco-PP-10	3.0	raco-PP-19	5.3
raco-PP-3	2.3	raco-PP-11	3.3	raco-PP-20	6.0
raco-PP-4	2.5	raco-PP-12	4.0	raco-PP-21	6.6
raco-PP-5	2.7	raco-PP-13	4.0	raco-PP-22	6.7
raco-PP-6	2.8	raco-PP-14	4.0	raco-PP-23	7.1
raco-PP-7	2.9	raco-PP-15	4.5		
raco-PP-8	3.0	raco-PP-16	4.5		

Table S7. ^{13}C NMR compositions of E/B-LLDPE, E/O-LLDPE, EPR and raco-PP samples utilized to validate the FP matching procedure with the proper FPE's.

	**E/B-LLDPE**	**E/O-LLDPE**	**EPR**	**raco-PP**
Sample #	x(B), mol%	x(O), mol%	x(E), mol%	x(E), mol%
V1	1.3	1.6	29.7	2.7
V2	1.3	1.6	36.4	2.7
V3	1.8	1.6	36.9	2.8
V4	2.2	1.65	37.1	2.8
V5	2.2	1.7	37.7	2.9
V6	2.3	2.2	38.0	3.0
V7	2.3	2.35	39.6	3.0
V8	2.3	2.4	44.7	3.1
V9	2.4	3.1	44.8	3.1
V10	2.4	3.2	44.9	3.1
V11	2.5	3.3	45.6	3.1
V12	2.5	3.6	47.1	3.2
V13	2.5	3.6	47.5	3.2
V14	2.6	3.9	50.5	3.2
V15	2.6	4.6	51.5	3.2
V16	2.7	4.8	53.3	3.3
V17	2.7	5.1	56.6	3.3
V18	2.9	5.4	57.9	3.4
V19	3.0	5.4	58.4	3.5
V20	3.2	5.8	60.2	3.7
V21	3.3	5.9	60.7	3.7
V22	3.4	6.2	60.9	4.1
V23	3.7	6.8	62.1	4.3
V24	4.3	7.6	62.9	4.3
V25	4.5	7.6	64.6	4.5
V26	4.9	7.9	64.8	4.5
V27	6.8	9.0	65.0	4.5
V28	7.6	9.2	65.8	4.6
V29	3.0	9.2	68.9	4.6
V30	3.0	9.2	69.2	4.8

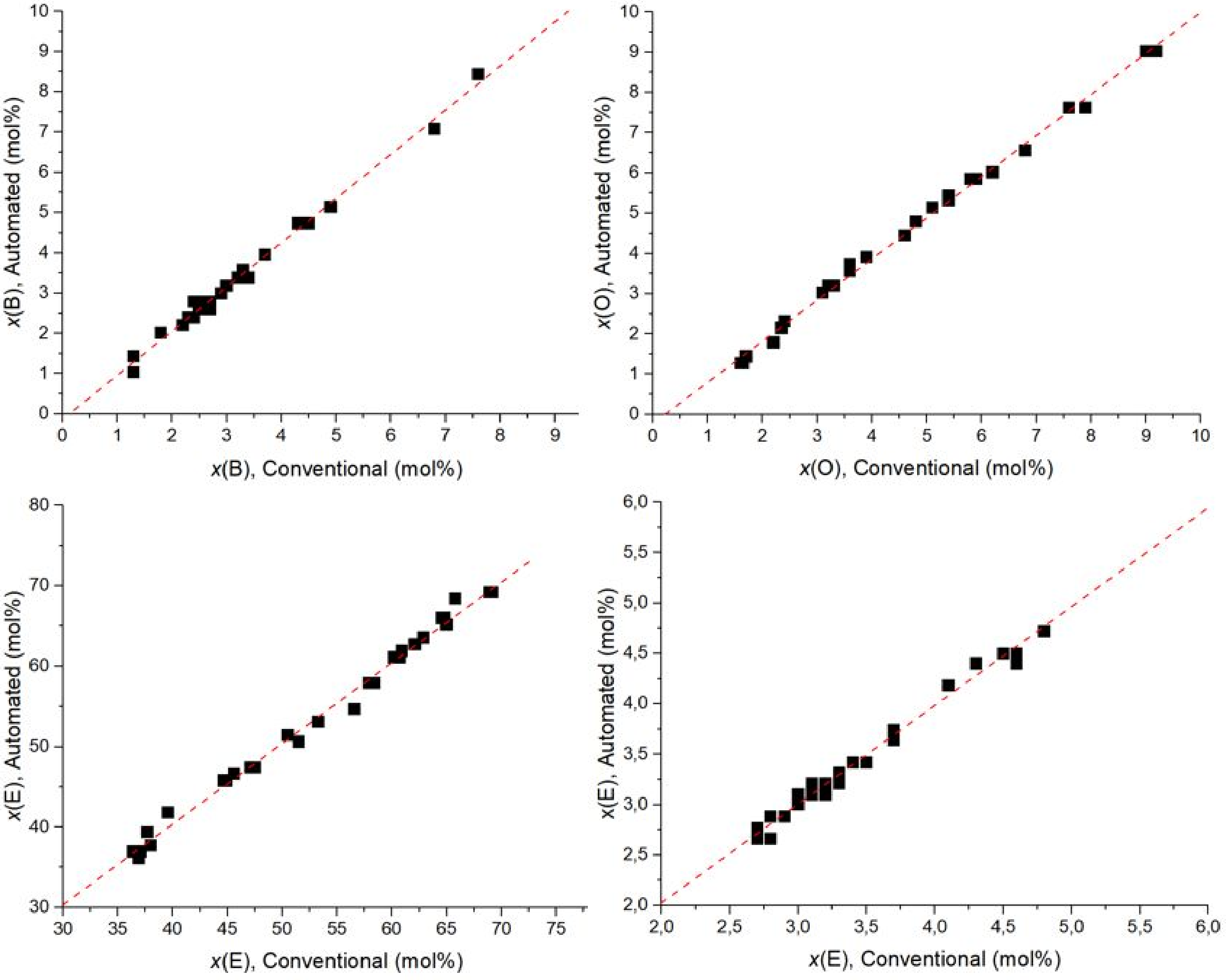

Figure S1. Correlation plots between conventional and automated ^{13}C NMR measurements of composition for the E/B-LLDPE (top left), E/O-LLDPE (top right), EPR (bottom left) and raco-PP (bottom right) samples of Table S7.
